# Colonization sites in carriers of ESBL-producing Gram-negative bacteria

**DOI:** 10.1186/s13756-018-0344-y

**Published:** 2018-04-12

**Authors:** Joffrey van Prehn, Anna M. Kaiser, Suzanne D. van der Werff, Rosa van Mansfeld, Christina M. J. E. Vandenbroucke-Grauls

**Affiliations:** Department of Medical Microbiology and Infection Control, VU university medical center, PK 1 X 124, P.O. Box 7057, 1007 MB Amsterdam, the Netherlands

**Keywords:** Screening, Colonization, ESBL, Extended-Spectrum Beta-Lactamase-producing Gram-negative bacteria, Infection control

## Abstract

**Objective:**

The distribution of Extended-Spectrum Beta-Lactamase-producing Gram-negative bacteria (ESBL-GNB) colonization sites is relevant for infection control guidelines on detection and follow-up of colonization. We questioned whether it is possible to rely solely on rectal swab culture for follow-up of ESBL-GNB colonization.

**Methods:**

We retrospectively assessed ESBL-GNB colonization sites in patients in a tertiary hospital in the Netherlands. The Laboratory Information Management System was queried for all bacterial cultures obtained between January 2012 and August 2016. All patients with one or more cultures positive for ESBL-GNB were identified and the distribution of ESBL-GNB positive sample sites was assessed. A subgroup analysis was performed on patients for whom at least one rectal swab specimen was available.

**Results:**

We identified 1011 ESBL-GNB carriers with 16,578 specimens for analysis. ESBL-GNB were most frequently isolated from the rectum (506/1011), followed by the urogenital (414/1011) and respiratory tract (142/1011), and pus (136/1011). For 588 patients at least one rectal swab specimen was available. In this subgroup, ESBL-GNB colonization was detected only in the rectum in 55.4% (326/588) of patients, in 30.6% (180/588) in the rectum and a different culture site, and in 13.9% (82/588) no rectal colonization was detected.

**Conclusions:**

Rectal colonization with ESBL-GNB was detected in 86% of ESBL-GNB carriers. However, in 14% of ESBL-GNB carriers we did not detect rectal colonization. Therefore, samples taken for follow-up of colonization with multi-drug resistant Gram-negative bacteria (MDR-GNB) should ideally also include samples from the site where the MDR-GNB was initially found.

## Introduction

Infection control guidelines give clear recommendations for the detection of colonization with multidrug-resistant Gram-negative bacteria (MDR-GNB) [1, 2]. Recommendations regarding type of screening cultures for the follow-up of colonization are less well defined [1, 2]. For example, the ESCMID guideline for infection control measures to reduce transmission of MDR-GNB in hospitalized patients strongly recommends active surveillance cultures in an epidemic setting, using stool or rectal or perirectal swab samples as well as samples from the inguinal area and manipulated sites [1]. However, no recommendations are given regarding follow-up of MDR-GNB colonization. The Dutch guideline for laboratory detection of highly resistant microorganisms refers to recommendations for carriers of *Salmonella* spp., which suggests that rectal cultures have to be used for follow-up [2]. For effective screening and surveillance programs it is important to have insight on the distribution of MDR-GNB colonization sites. However, literature on this topic is scarce, few articles explicitly focus on colonization sites, and the number of patients analysed in these studies is limited. We questioned whether it is possible to rely on the rectum as the single site to obtain cultures for follow-up of MDR-GNB colonization. To this end we retrospectively investigated the distribution of colonization sites in patients colonized with Extended-Spectrum Beta-Lactamase-producing Gram-negative bacteria (ESBL-GNB), and assessed the value of rectal swab culture in a subgroup that had at least one rectal swab culture available.

## Methods

### Setting

In our tertiary teaching hospital, we have an active screening policy for MDR-GNB, which includes ESBL-producing Gram-negative bacteria. On admission, patients with risk factors for colonization with multidrug-resistant bacteria (e.g. having been admitted to a hospital outside the Netherlands within the prior 2 months), are screened for MDR-GNB by rectal culture and when applicable urinary culture (when a urinary catheter is in situ), sputum culture (when the patient is intubated), and wound cultures (in case of open wounds). Patients with known MDR-GNB colonization are nursed with contact precautions and on readmission they are screened by collecting swabs of the rectum and the site where the MDR-GNB was initially found. On the intensive care units and haematology ward selective decontamination of the digestive tract (SDD) is used and active screening cultures are obtained according to standardized protocols: on admission and twice a week thereafter.

### ESBL detection

The ESBL-producing Gram-negative bacteria in this study include Enterobacteriaceae and *Pseudomonas* and *Acinetobacter* spp. Criteria for screening for ESBL production were applied according to the Dutch Society for Medical Microbiology guideline (which is largely based on CLSI and EUCAST guidelines): positive ESBL screening was defined as a MIC > 1 mg/L for cefotaxime (or ceftriaxone) and/or ceftazidime [2]. Phenotypic ESBL confirmation was performed with combination disk diffusion testing. For group 1 Enterobacteriaceae in which chromosomal AmpC beta-lactamases are uncommon or absent (*Escherichia coli*, *Klebsiella* spp., *Proteus mirabilis*, *Salmonella* spp. and *Shigella* spp.) confirmation is positive when the zone around the disk/tablet with either cefotaxime or ceftazidime is ≥5 mm larger with clavulanic acid than without; for group 2 Enterobacteriaceae in which chromosomal AmpC beta-lactamases are common (*Citrobacter freundii, Enterobacter* spp. *Hafnia alvei, Morganella morganii, Providencia* spp. *and Serratia* spp.*)* confirmation is positive when the zone around the disk/tablet with cefepime is ≥5 mm larger with clavulanic acid than without [2]. ESBL production in non-fermenters was also confirmed by cefepime combination disk diffusion testing.

### Data query

We queried our Laboratory Information Management System for all bacterial cultures between January 2012 and August 2016 to identify all patients with one or more cultures positive for ESBL-GNB. All cultures of patients included in the analysis were studied, which include both screening and diagnostic specimen cultures.

### Subgroup analysis

To assess the value of rectal swab culture we performed a subgroup analysis on patients for whom at least one rectal swab specimen was available. This subgroup was further divided in patients that had been admitted to the ICU or haematology wards (where SDD is used) at least once, and patients that had never been admitted to one of these wards.

## Results

### Overall analysis

We identified 1011 patients with one or more cultures positive for ESBL-GNB, which yielded 16,578 specimens for analysis. Per patient a median of 9 samples (inter quartile range 4 – 20) were obtained, the median interval between the first and the last sample obtained was 88 days (interquartile range 8 - 518). The distribution of colonization sites in these patients is shown in Table 1.

**Table 1:** Distribution of ESBL-GNB colonization sites. Overall data for all patients colonized with Extended-Spectrum Beta-Lactamase-producing Gram-negative bacteria (*n* = 1011)

Sample site/type	N of patients colonized (N_total_ = 1011)	%
Rectum	506	50,0
Urogenital tract	414	40,9
Respiratory tract & Pleural aspirate	142	14,0
Pus	136	13,5
Blood & Intravenous or arterial catheter tip	75	7,4
Other	60	5,9
Ascites/Bile/Drain/Feces	40	4,0
Biopsy & Aspirate	22	2,2
Ear & Nose	4	0,4
Joint prosthesis	3	0,3
Central Nervous System	1	0,1

### Subgroup analysis

**Fig. 1:**
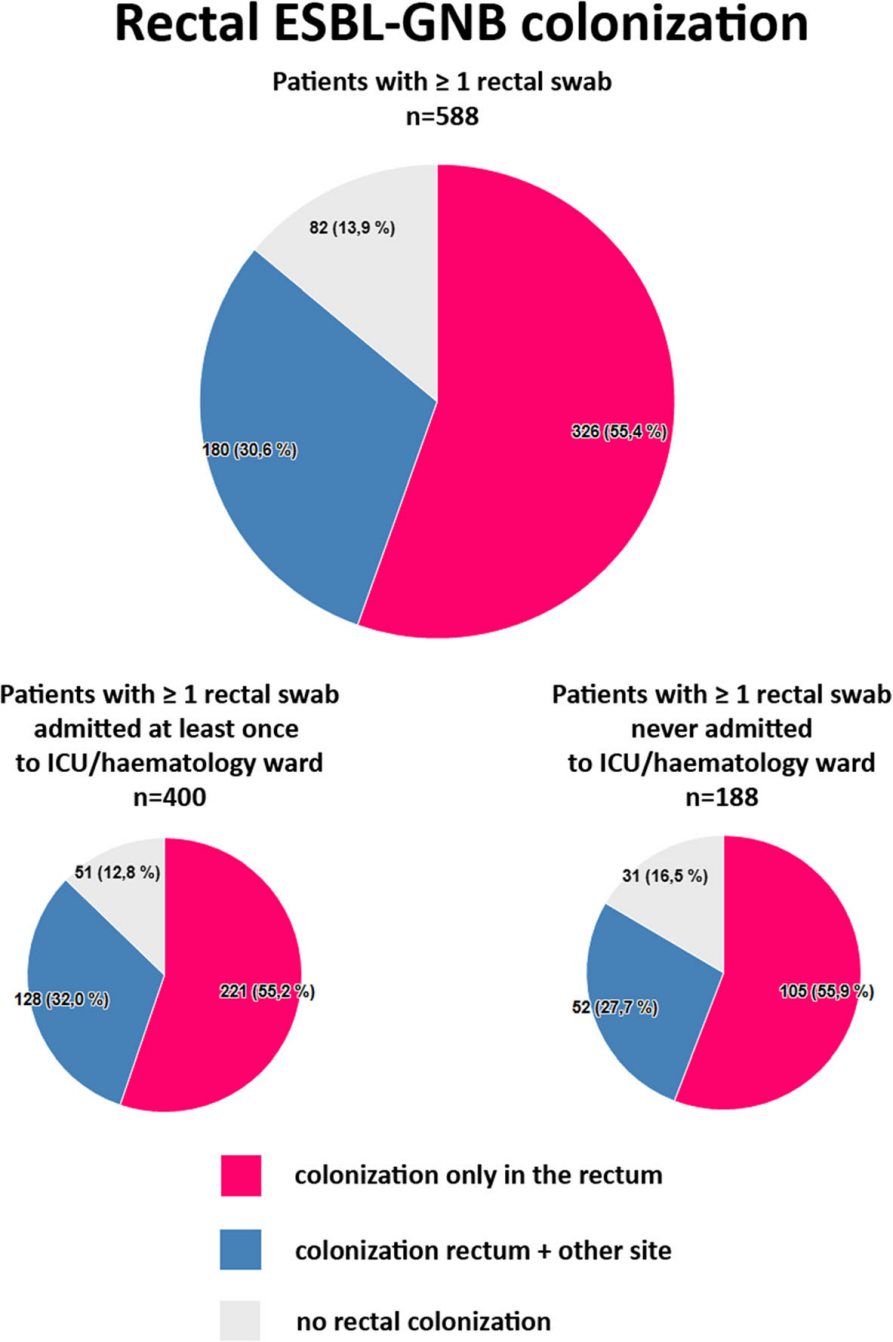
Pie charts of rectal ESBL-GNB colonization in a subgroup of patients with at least one rectal swab available. Analysis of the subgroup as a whole and of the groups that were either admitted at least once or were never admitted to the ICU or haematology ward (where SDD is used)

For 588 patients at least one rectal swab specimen was available (totalling 12,436 specimens). In this subgroup, colonization was detected in the rectal swab culture only in 326 of 588 ESBL-GNB carriers (55.4%). In 82 patients (13.9%) rectal swab cultures remained negative. Colonization was detected both in the rectum and at a different culture site in 180 patients (30.6%). The distribution of colonization sites in these patient groups is shown in Table 2. Pie charts that illustrate rectal colonization in the subgroup as a whole are shown in Fig. 1. This figure also depicts rectal colonization in the groups that were either admitted at least once, or were never admitted to the ICU or haematology ward. The 82 ESBL-GNB carriers in whom the colonization was never detected in a rectal culture had on average 6.3 rectal swab specimens with negative culture for ESBL-GNB. In 39% of these patients the maximum interval between a positive culture from another site and a negative rectal culture was 7 days. Of the 506 ESBL-GNB carriers in whom rectal colonization was detected, there were 28 patients that had a negative rectum sample while a prior and subsequent sample was positive.

**Table 2:** Distribution of ESBL-GNB colonization sites in a subgroup of patients with at least one rectal swab available

Sample site/type	Colonization rectum only (*N* = 326)	%	Colonization rectum + other site (*N* = 180)	%	No colonization rectum (*N* = 82)	%	Total (*N* = 588)	%
Rectum	326	100,0	180	100,0	–	–	506	86,1
Respiratory tract & Pleural aspirate	–	–	93	51,7	25	30,5	118	20,1
Urogenital tract	–	–	72	40,0	33	40,2	105	17,9
Pus	–	–	47	26,1	21	25,6	68	11,6
Blood & Intravenous or arterial catheter tip	–	–	29	16,1	9	11,0	38	6,5
Other	–	–	29	16,1	3	3,7	32	5,4
Ascites/Bile/Drain/Feces	–	–	13	7,2	6	7,3	19	3,2
Biopsy & Aspirate	–	–	11	6,1	2	2,4	13	2,2
Ear & Nose	–	–	2	1,1	1	1,2	3	0,5
Central Nervous System	–	–	1	0,6	–	–	1	0,2
Joint prosthesis	–	–	1	0,6	–	–	1	0,2

Data for all patients included in the subgroup-analysis (*n* = 588) and data stratified per patient group: rectal colonization only (55,4%), rectum plus another site colonized (30,6%), and no rectal colonization (13,9%)

## Discussion

In this study we detected rectal colonization in 86% of patients colonized with ESBL-GNB and in 14% of ESBL-GNB carriers, no rectal colonization was detected. These 14% of ESBL-GNB carriers might potentially be missed if followed with rectal cultures only. This finding is in line with the scarce literature available on ESBL colonization. Papst et al. found that in 114 patients, 10% of those with positive sample sets had negative rectal swabs [3]; Tschudin-Sutter et al. found rectal colonization in only 69% of 133 patients [4].

Of course, our results are limited by the retrospective nature of the analysis. However, the standardized protocols for ESBL-GNB detection and follow-up at our centre and the relatively large sample size add to the validity of the retrospective analysis. Ideally, rectal samples would have been taken simultaneously with positive ESBL-GNB samples from non-rectal body sites. Therefore we have studied the number of negative rectal swab cultures and interval between an ESBL-GNB positive culture and negative rectal swab culture in the colonized patients without apparent rectal colonization. Thirty-nine percent of patients had negative rectal cultures taken within 8 days of an ESBL-GNB positive sample at another location: loss of colonization within this interval seems not likely. This finding might partly overcome the objection that the absence of rectal colonization in 14% of ESBL-GNB carriers might be caused by sampling bias. In patients with longer intervals between negative rectal samples and ESBL-positive samples from non-rectal sites, natural loss of colonization, the use of antibiotics and/or SDD might have led to negative rectal samples. However, we found similar rectal colonization rates in patients that presumably received SDD (e.g. were admitted to the ICU or haematology ward at some point in the study period), compared to patients that presumably did not receive SDD (e.g. were never admitted to the ICU or haematology ward during the study period). Although this study this has its limitations, we feel that our data can be of help to formulate infection control guidelines. Data on MDR-GNB colonization sites is scarce; more insight in colonization sites is urgently needed, and in the absence of large prospective studies, retrospective analysis with considerable sample sizes are welcomed. Future prospective trials should ideally use standardized sample protocols that will limit the time between rectal sampling and positive ESBL-GNB cultures at non-rectal sites. It is also advisable that such trials would focus on the cost-effectiveness of screening protocols.

It is of interest that we found that 28 of 506 patients with positive rectal cultures had a negative rectal sample obtained between two positive rectal samples. This could represent either loss of colonization or sampling error. The latter is the reason why in the Netherlands we require a patient to have two consecutive samples negative for MDR-GNB, before isolation precautions can be discontinued. However, when implementing MDR-GNB screening protocols the potential gain of extra cultures (higher sensitivity, less potential ESBL spreading) has to be balanced against the economic impact of performing more cultures. In addition, local and national screening protocols should be adapted to the local and national MDR-GNB epidemiology. In the Netherlands the prevalence of ESBL-producing Enterobacteriaceae is estimated to be around 10% in clinical isolates [5]. In areas with a higher prevelance of ESBL-GNB it might be more cost-effective to direct MDR-GNB screening programs at carbapenemase producing bacteria. In addition, in more resource-limited countries the potential gain of extra cultures might be outweighed by the extra-costs of performing more cultures. Furthermore, we do not intend to recommend repeating invasive procedures for screening purposes.

In this study we questioned whether our current practice of MDR-GNB follow-up by rectal cultures plus a culture from the site where the MDR-GNB was initially found is more sensitive than follow-up by rectal cultures only. In conclusion, our data and the current available evidence indicate that 10 to 30% of colonized patients do not carry ESBL-GNB in the rectum [3, 4]. This finding should have implications for screening and follow-up programs of MDR-GNB colonization: ideally, screening and follow-up programs of MDR-GNB colonization should not solely rely on rectal cultures but should preferably also include the site where the MDR-GNB was initially found.
